# Intra-Host Diversity and Emergence of Unique GBV-C Viral Lineages in HIV Infected Subjects in Central China

**DOI:** 10.1371/journal.pone.0048417

**Published:** 2012-11-12

**Authors:** Haoming Wu, Abinash Padhi, Junqiang Xu, Xiaoyan Gong, Po Tien

**Affiliations:** 1 College of Chemistry and Molecular Sciences, Wuhan University, Wuhan, Hubei, China; 2 College of Life Sciences, Wuhan University, Wuhan, Hubei, China; 3 Department of Biology, The Pennsylvania State University, University Park, Pennsylvania, United States of America; 4 Hubei Provincial Centers for Disease Control and Prevention, Wuhan, Hubei, China; 5 Institute of Microbiology, Chinese Academy of Sciences, Beijing, China; University of Sao Paulo, Brazil

## Abstract

GB virus C (GBV-C), which is highly prevalent among HIV/AIDS, seemed to slow the HIV disease progression. The HIV/GBV-C co-infected individuals may represent an interesting model for the investigation of the role played by HIV infection and/or the immune system in driving the evolution of the GBV-C viral populations. The present study investigated the prevalence and population dynamics of GB virus C in HIV infected individuals representing 13 geographic regions of Hubei Province of China. Approximately 37% of HIV-1 infected individuals were infected with GBV-C and genotype 3 is appeared to be predominant. Utilizing the 196 complete E2 nucleotide sequence data from 10 HIV/GBV-C infected individuals and employing coalescence based phylogenetic approaches; the present study has investigated the intra-host dynamics of GBV-C. The results revealed patient-specific unique GBV-C viral lineages and each viral lineage showed the evidence of rapid population expansion in respective HIV-1 infected patients, thus suggesting HIV-1 was unlikely to have been inhibiting effect on the GBV-C viral replication. GBV-C in all patients has experienced intense purifying selection, suggesting the GBV-C viral invasion and subsequent expansion within the HIV-1 infected hosts without any modification of the functional epitopes at their membrane protein. The finding of within host GBV-C recombinant sequences indicated recombination was one of the significant forces in the evolution and divergence of GBV-C.

## Introduction

GB virus C (GBV-C), a single stranded and positive sense RNA virus of the family *Flaviviridae*, has worldwide distribution in the general population. Approximately 5% and 5–18% of healthy blood donors in developed [1] and developing countries [2], [3], [4] were GBV-C viraemic. However, the prevalence of GBV-C in HIV-1 infected populations was reported to be 17–41% [5], [6], [7], [8], [9], [10], [11]. Previous studies have reported that individuals co-infected with HIV/GBV-C had a delayed CD4^+^ T cells depletion, lower HIV viral loads, and delayed progression of HIV disease to AIDS [7], [11], [12], [13], [14], [15]. Thus, these clinical studies suggested persistent GBV-C viremia significantly improved survival in HIV-1 infected populations [16], [17]. In order to understand the role or the influence of GBV-C, knowledge of the GBV-C viral dynamics in HIV-infected individuals is therefore, crucial.

Phylogeny-based analysis suggested the existence of seven GBV-C genotypes with worldwide distribution [18]. Although GBV-C genotypes 1, 2, 3, 4, 5, and 6, respectively, are predominant in West Africa, Europe & North America, parts of Asia including China and Japan [19], [20], Southeast Asia [21], South Africa [22], and in Indonesia [23], a newly designated genotype, i.e., genotype 7 has recently been identified in China [18]. These reports suggested an extent of geographic specificity to the GBV-C viral genotypes. The appearance of multiple GBV-C genotypes has led the researchers to suggest that differences in GBV-C strains circulating within population might impact HIV disease differently [24], [25], [26].

Due to its unique host-pathogen interaction and higher evolutionary rate, GBV-C has been proposed to be the potential genetic marker to track the ancient human migrations [27], [28]. In addition, recent reports on its role in suppressing the HIV-1 infection [7], [11], [12], [13], [14], [15] also warranted for a detailed understanding of the dynamics of GBV-C viral emergence within individual hosts. Utilizing the complete coding E2 gene sequence data, the objective of the present study was to investigate the population dynamics, the patterns of genetic polymorphisms, and the role of natural selection and recombination in the GBV-C viral evolution and emergence within the HIV infected individuals.

## Materials and Methods

### Serum Samples, RNA Extraction, and GBV-C Detection

**Figure 1:**
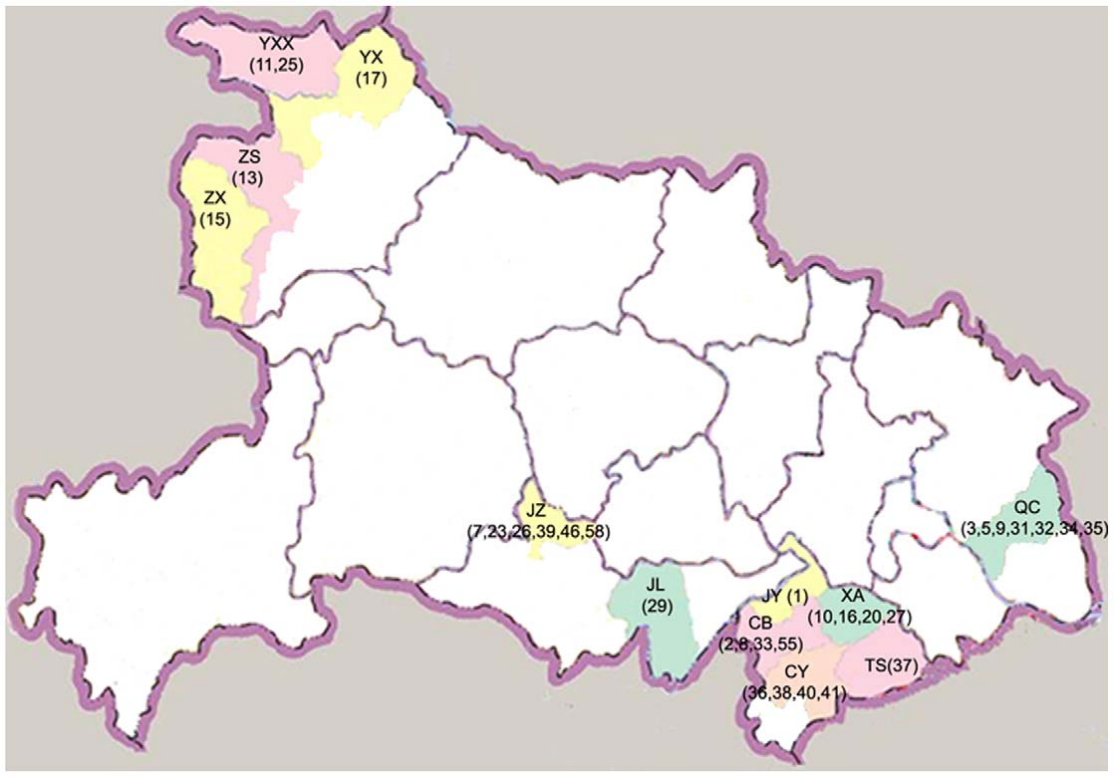
Geographic origin of samples in Hubei Province, China.

The samples used in this study were obtained from Hubei Provincial Center for Disease Control and Prevention. One hundred and fifty-six HIV-1 positive samples were collected between October 2009 and November 2010, and subjected to GBV-C RNA detection. All patients representing 13 different geographic regions (Qichun, Jingzhou, Yunxian, Yunxixian, Zhushan, Zhuxi, Jianli, Jiayu, Chibi, Xianan, Tongshan, Tongcheng, Chongyang) were under the care of public outpatient services from Hubei province in China (Fig. 1), with a median CD4 cell count of 313 cells/µl, the HIV load of most of them was under detection baseline.

Total RNA was extracted from 100 µl serum for each patient using the Trizol LS reagents (Invitrogen, Carlsbad, California, USA) following the manufacturer's instructions. The quantity of 2 µg of extracted RNA was reverse transcribed using random hexamers (Promega, Madison, Wisconsin, USA), M-MLV reverse transcriptase (Promega, Madison, Wisconsin, USA) and ribonuclease inhibitor (Biostar International, Canada) in a total volume of 25 µl for 60 min at 37°C. A fragment of 208 bp of 5′ untranslated region (5′-UTR) of the GBV-C was amplified by nested PCR using primers 5′-UTR-F1/R1 (outer) and 5′-UTR-F2/R2 (inner) (Table 1) [2]. The PCR reaction was initiated with a preheating procedure (95°C for 5 min) and performed on a thermocycler (Eppendorf, Germany) for 30 cycles (consisting of denaturation at 94°C for 1 min, annealing at 55°C for 30 s and extension at 72°C for 30 s) and a final extension cycle at 72°C for 10 min. The PCR product was submitted to electrophoresis analysis on 1.0% agarose gel, stained with ethidium bromide and visualized under UV illumination.

**Table 1:** Primers used for GBV-C detection and genotyping.

	Primer	Polarity	Sequence^a^	Position^b^	Amplicon length (bp)
5′-UTR	UTR-F1	outer, forward	5′-CAGGGTTGGTAGGTCGTAA ATCC-3′	130–152	208
	UTR-R1	outer, reverse	5′-CCTATTGGTCAAGAGAGACAT-3′	351–371	
	UTR-F2	inner, forward	5′-GGTCAYCYTGGTAGCCACTATAGG-3′	154–177	
	UTR-R2	inner, reverse	5′-AAGAGAGACATTGWAGGGCGACGT-3′	338–361	
E2	E2-F	outer, forward	5′-RGTGGGRRAGTGAGTTTTGGAGAT-3′	961–984	1242
	E2-OR	outer, reverse	5′-GCCTCHGCCAGCTTCATCAGRTA-3′	2214–2236	
	E1fcon	inner, forward	5′-TGGGAAAGTGAGTTTTGGAGATGG-3′	963–986	
	E2-IR	inner, reverse	5′-AAAYACAAARTCCARVAGCARCCA-3′	2181–2204	

a Mixed base code Y was used for the mixture of C and T; W for A and T; R for A and G; H for A, T and C; V for G, A and C; D for G, A and T.

b Nucleotide positions are numbered as for AF121950.

### Amplification, Cloning, and Sequencing

The 1242 bp length of GBV-C including partial of E1 gene and entire E2 gene (positions 963–2204 of the AF121950) from 10 HIV/GBV-C dual infection patients was amplified using Pyrobest DNA Polymerase (Takara, Japan). To examine PCR error from the DNA polymerase, a known sequence from empty vector pcDNA3.1 was PCR amplified, cloned and sequenced under identical conditions. Analysis of 10 independent clones showed absolute identity with the parental sequence. Then, the amplification of GBV-C E2 gene was performed by nested PCR using E2_F/OR (outer) and E1fcon/E2_IR (inner) primers (Table 1) [29]. The touchdown PCR reaction was initiated with a preheating procedure (95°C for 5 min) and performed on a thermocycler for 30 cycles (the annealing temperature was progressively lowered from 65°C to 50°C by 1°C every cycle, followed by 15 additional cycles at 50°C) and a final extension cycle at 72°C for 10 min. Subsequently, PCR products were extracted from the gel using Easy Pure Quick Gel Extraction Kit (TransGen Biotech, Beijing, China) and then were TA-cloned into plasmid pTA2 vector using the Target Clone™ kit (Toyobo, Osaka, Japan) following the manufacturer's instructions. After an incubation period of 24 h, single clones from each plate were randomly selected based on the color reaction using Xgal-IPTG system and grown in LB broth in the presence of 50 µg/ml ampicillin. Twenty clones from each patient were collected and sequenced. Sequencing was carried out by use of the ABI-PRISM3730 sequencer in Sangon Biotechnology Company, China.

### Detection of Anti-GB virus C E2 antibody

The determination of antibodies to the GBV-C E2 protein in serum samples was performed by using the human GBV-C E2 Elisa kit (R&D Systems, Minneapolis, USA), in accordance with the manufacturer's instructions.

### Genotype Determination

A total of 196 complete E2 nucleotide coding sequences representing 10 HIV/GBV-C co-infected patients were aligned using MEGA4.1 [30]. All the sequences generated in this study were deposited in GenBank with accession numbers **JX458516–JX458711**. To determine the genotype affiliation of each sequence, reference sequences representing all the seven previously defined genotypes were retrieved from GenBank and were included in the phylogenetic analysis. The neighbor-Joining tree was reconstructed under the maximum composite likelihood model implemented in MEGA. Using the same program the nodal supports were determined with 1000 bootstrap replicates.

### Within Host Evolutionary Dynamics

Full length E2 sequence data were utilized to estimate molecular diversity indices, mismatch analysis, Tajima's D, Fu's F, and to reconstruct the Bayesian skyline plots. Prior to these analyseis, six different recombination detection methods implemented in RDP3 software package [31] were used to test whether there was any evidence of recombination. The individual programs RDP [32], GENECONV [33], Bootscan [34], Maximum Chi [35], Chimaera [36], SiScan [37] and 3Seq [38], were implemented for the analysis. The recombinant sequences were excluded from the analysis.

Arlequin ver 3.5 [39] was used for the estimation the molecular diversity indices such as nucleotide (π) diversities, the mean number of pairwise differences (d), Tajima's *D* statistic [40] and Fu's F_S_ statistic [41] and to compute the frequency of pairwise differences to evaluate the hypothesis of sudden expansion [42]. The validity of expansion hypothesis was tested using a parametric bootstrap approach by simulations of 10,000 random samples [43].

A Bayesian MCMC approach under the clock model as implemented in BEAST ver. 1.6.2 [44] was used to determine the time to the most recent common ancestor (TMRCA) of the GB virus C in each patient. A rate of 3.9×10^−4^ nucleotide substitutions per site per year, previously reported for GBV-C was used [45]. Phylogenies were evaluated using a chain length of 20 million states under HKY+G4. In each case, MCMC chains were run for sufficient time to achieve convergence. Uncertainty in the data was described by 95% high-probability density (HPD) intervals. Convergence of trees was checked using Tracer v1.5 (available at: http://beast.bio.ed.ac.uk/Tracer). The inferred trees were visualized using FigTree ver. 1.3.1 (available at: http://tree.bio.ed.ac.uk/software/figtree/). We utilized the Bayesian skyline plot (BSP) as a coalescent prior to inferring the population dynamics of GBV-C within the HIV infected individual. We randomly selected 10 HIV infected patients representing different geographic region of Hubei province and performed the Bayesian coalescent analysis on each set of sequences representing each patient and evaluated the BSP patterns. The estimated population size reflects the effective population size of GBV-C in each patient. Therefore, the unit of BSP should be the viral effective population size through time.

To determine the putative role of positive selection (ω>1) in the GBV-C viral diversity within each patient, we performed site-specific positive selection analysis using Fixed- Effect Likelihood (FEL) via the Datamonkey web server [46]. Site with P-value<0.05 were considered to be under positive selection. The ML approach implemented in CODEML of PAML package version 3.15[47] was also used to detect the sites under positive selection in each patient. The codon-based substitution models (M7, M8) implemented in the CODEML allows the dN/dS to vary among sites. The likelihood ratio test (LRT) was used to compare M7 model that assume no positive selection (dN/dS<1) with the M8 model that assume positive selection (dN/dS>1). Sites with Bayes Empirical Bayes (BEB) posterior probabilities >95% were considered to be under positive selection.

## Results

### GBV-C Infection Status

A total of 156 HIV-1 positive samples were collected in 13 prefectures of Hubei province of China. Transmission risk factors for the infection with GBV-C were deduced from the viral prevalence in the HIV risk groups. Heterosexual promiscuity (59.6%) was the main risk factors in our patients, while the remaining patients had a history of blood transfusion (17.5%), male homosexual promiscuity (15.8%) or injection drug abuse (5.3%). Only one out of 57 patients was the vertical transmission of HIV from infected mother to infant. All samples were tested for the presence of GBV-C RNA using primers from the 5′-UTR. Fifty seven cases of active GBV-C infections were identified, resulting in a prevalence of 36.5% GBV-C among the HIV-1 infected subjects in Hubei province. Among those tested as positive for GBV-C RNA, only patient QC_5 was detected anti-E2 antibody positive, others were anti-E2 antibody negative. Of the total 57 dual-infected patients, 36 (63.2%) were males and 21(36.8%) females, 38 (66.7%) patients were on Highly Active Anti-Retroviral Therapy (HAART), and the others were untreated.

### Phylogenetic analysis

**Figure 2:**
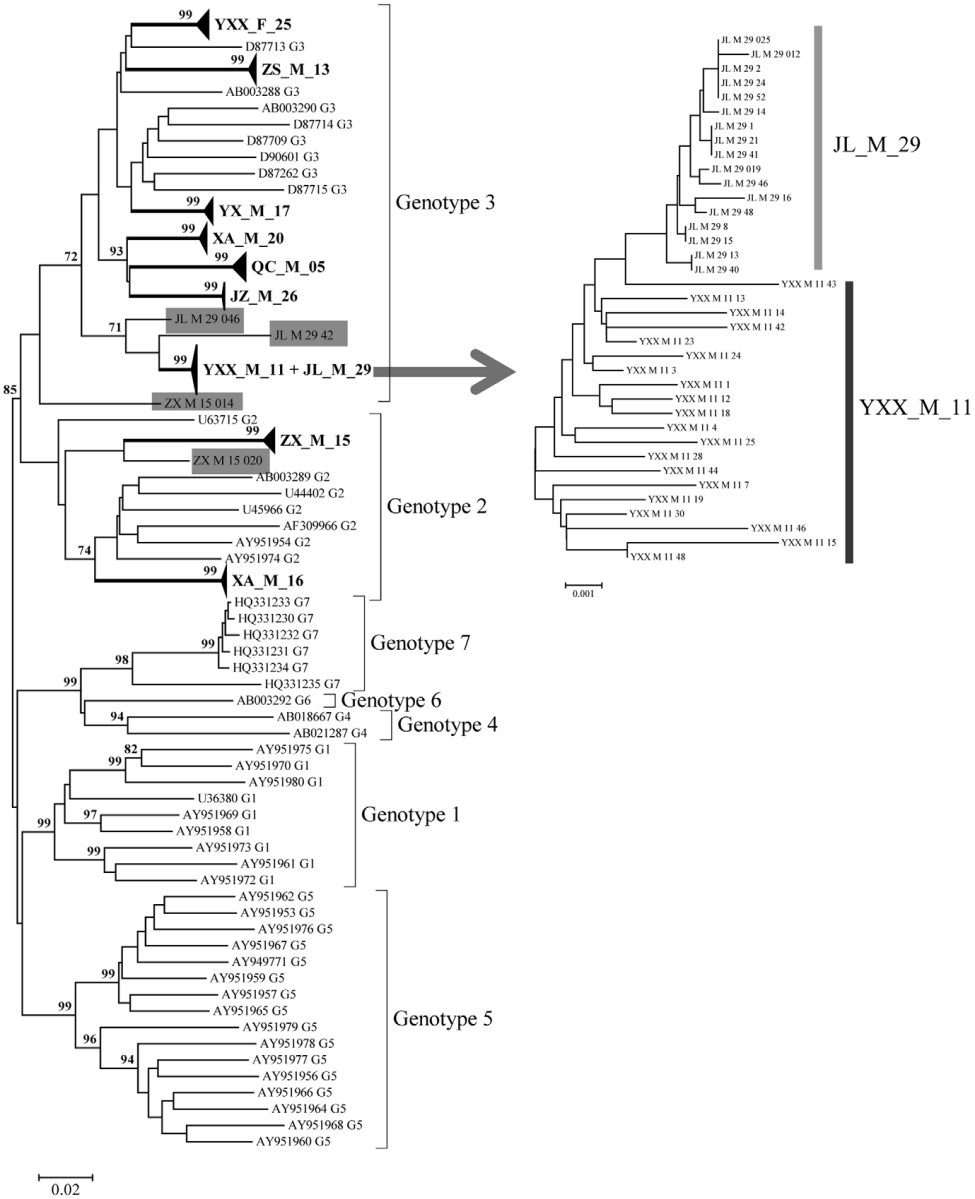
Phylogenetic tree inferred from the complete E2 sequence data showing GBV-C variants in each HIV-infected subjects formed a unique cluster and emerged as a unique lineage with strong statistical support. Sequences representing each genotype were used as references for genotype identification. Sequences with GenBank accession numbers were the reference sequences. Isolates shaded in grey colors were the recombinant sequences (Table 2). Patients YXX_M_11 and JL_M_29 together formed a unique cluster. All the variants of JL_M_29 clustered together and appeared to emerge from a single GBV-C variant of YXX_M_11. GBV-C in patients QC_M_5, XA_M_20, and JZ_M_26 appeared to be monophyletic and therefore shared the common ancestor. Bootstrap support ≥70 were shown at the base of the node. Each patient was coded with geographic region, sex, and a unique patient number.

Prior to the genetic analysis, we performed six different recombination detection tests to identify whether any of the cloned sequences were recombinant. Four sequences, two from patient ZX_M_15 and the others from patient JL_M_29, were recombinant (Table 2; Fig. 2). Therefore, these recombinant sequences were excluded from further genetic analysis. To evaluate the possible emergence of recombinant sequences, we performed the PCR based experiment by mixing two isolates representing different genotypes. GBV-C E2 clone QC_5_21 (genotype III) and XA_16_001 (genotype II) were physically mixed with the same ratio to use as a template and the E2 gene was PCR amplified, cloned and sequenced under identical conditions. Recombination analysis on those PCR-base recombinant sequences showed there were three recombinant sequences in a total of 10 clones. However, 4 recombinant sequences were detected in a total of 196 E2 sequences. Nevertheless, these results are consistent with the fact that recombination in natural population is less frequent than in the experimental condition [48].

**Table 2:** Detection of recombination in complete E2 sequences by six different methods.

Recombination Event Number	Breakpoint Positions^a^	Recombinant Sequence(s)	RDP	GENECONV	Maxchi	Chimaera	SiSscan	3Seq
1	636-32	ZX_M_15_014	6.33E-19	3.60E-13	1.04E-13	1.37E-13	4.09E-17	6.53E-23
2	1106-493	ZX_M_15_020	8.61E-10	1.18E-09	1.96E-13	3.98E-08	6.61E-13	1.80E-05
3	662 - 1106	JL_M_29_42	4.95E-12	1.44E-08	1.70E-09	1.28E-09	2.57E-11	3.38E-22
4	536 - 54	JL_M_29_046	NS	7.35E-06	8.92E-11	5.79E-11	1.14E-14	NS

a Breakpoint Positions Relative to U36380.

NS: Not significant at p = 0.0005.

Phylogenetic analysis has revealed that while eight HIV patients were infected with GBV-C genotype 3, two patients were infected with GBV-C genotype 2 (Fig. 2). GBV-C E2 sequences from the respective patients formed a patient-specific unique cluster with strong bootstrap support (Fig. 2). GBV-C viral strains from patients XA_M_20, QC_M_05, and JZ_M_26 appeared to be monophyletic (Fig. 2). Although patients YXX_M_11 and JL_M_29 clustered together, GBV-C sequences from YXX_M_11 were basal to the GBV-C sequences from JL_M_29, indicating that the GBV-C in YXX_M_11 was likely the founding population for JL_M_29. The observation of low branching pattern (Fig. 2), low nucleotide diversity (π) (Table 3), and mean pairwise differences (d) (Table 3) in JL_M_29 further indicated that patient JL_M_29 was relatively recently infected and the viral population within JL_M_29 was emerged from a founding population (Fig. 2; Table 3).

**Table 3:** Infection route, therapy, number of clonal sequences, nucleotide diversity, mean nucleotide pairwise differences, mismatch distribution p-value, neutrality test (Tajimas'D and Fu's F), the nonsynonymous to synonymous substitutions, and the estimated time when each patient might have infected with GBV-C were mentioned.

Patients Code	Infection Route	Therapy	n	π	d	Mismatch -P	Tajimas'D	Tajimas'D P	Fu's F	Fu's F P	ω (95% HPD)	TMRCA in year (95% HPD)
JL_29	heterosexual	NO	17	0.001456±0.000988	1.808824±1.095936	0.167	0.06437	0.560	−5.9375	0.000	0.04 (0.02–0.07)	2008 (2005–2009)
XA_16	intravenous drug	HARRT	19	0.003076±0.001815	3.725146±1.967228	0.203	−0.4936	0.326	−11.637	0.000	0.14 (0.05–0.27)	2005 (2002–2007)
XA_20	heterosexual	HARRT	20	0.002924±0.001727	3.631579±1.920425	0.444	−1.6038	0.034	−10.997	0.000	0.20 (0.10–0.36)	2005 (2002–2007)
YXX_11	paid blood donor	NO	20	0.007043±0.003789	8.747368±4.213996	0.589	−1.8122	0.023	−13.602	0.000	0.08 (0.05–0.15)	2000 (1997–2003)
YXX_25	paid blood donor	HARRT	20	0.005653±0.003095	7.021053±3.442320	0.712	−2.1745	0.001	−15.734	0.000	0.05 (0.02–0.11)	2002 (2001–2005)
ZS_13	blood	NO	20	0.005301±0.002920	6.584211±3.246829	0.891	−1.0874	0.140	−8.194	0.003	0.13 (0.06–0.23)	2003 (1999–2006)
ZX_15	paid blood donor	NO	17	0.008618±0.004651	9.669118±4.662595	0.651	−1.7883	0.020	−9.5448	0.001	0.04 (0.03–0.06)	1999 (1994–2002)
YX_17	heterosexual	NO	18	0.005519±0.003048	6.849673±3.382386	0.777	−2.0025	0.009	−13.369	0.000	0.11 (0.06–0.19)	2002 (1999–2005)
JZ_26	homosexual	HARRT	20	0.003307±0.001941	3.789474±1.991743	0.624	−2.2332	0.001	0.26629	0.555	0.05 (0.01–0.16)	2006 (2002–2008)
QC_5	heterosexual	NO	20	0.008860±0.004695	10.99473±5.217300	0.671	1.54914	0.950	−3.4866	0.065	0.06 (0.03–0.13)	1996 (1990–2001)

n: Number of sequences.

π: Nucleotide diversity.

d: Mean Paidwise difference.

P: P-value.

HPD: Highest Posterior Density.

ω: Rate of Nonsynonymous (dN) to the Rate of Synonymous (dS) substitutions.

### Within-host Population dynamics

**Figure 3:**
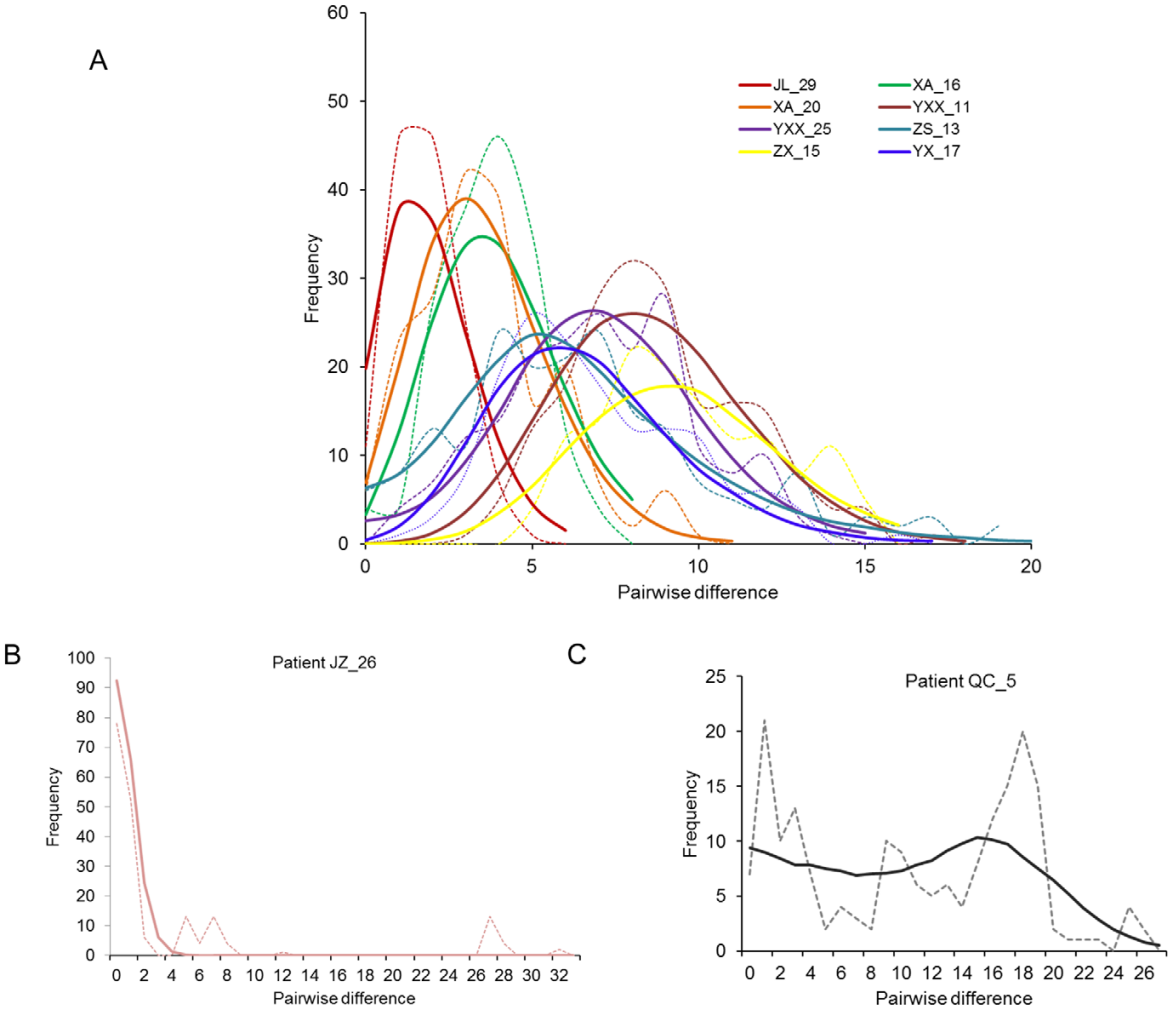
Distribution of the pairwise nucleotide sequence differences within each patient. (A) Eight patients showed unimodal distribution indicating sudden population expansion of GBV-C in respective individuals. (B) Patient JZ_26 virus showed an L-shape distribution. The L-shape distribution in JZ_26 was sign of post bottleneck population expansion, (C) Patient QC_5 showed multiple peaks. The hypothesis of sudden population expansion for each patient could not be rejected (p>0.05, Table 3). The observed and simulated pairwise differences are shown in dotted and solid lines, respectively. Recombinant sequences were excluded from the analysis.

**Figure 4:**
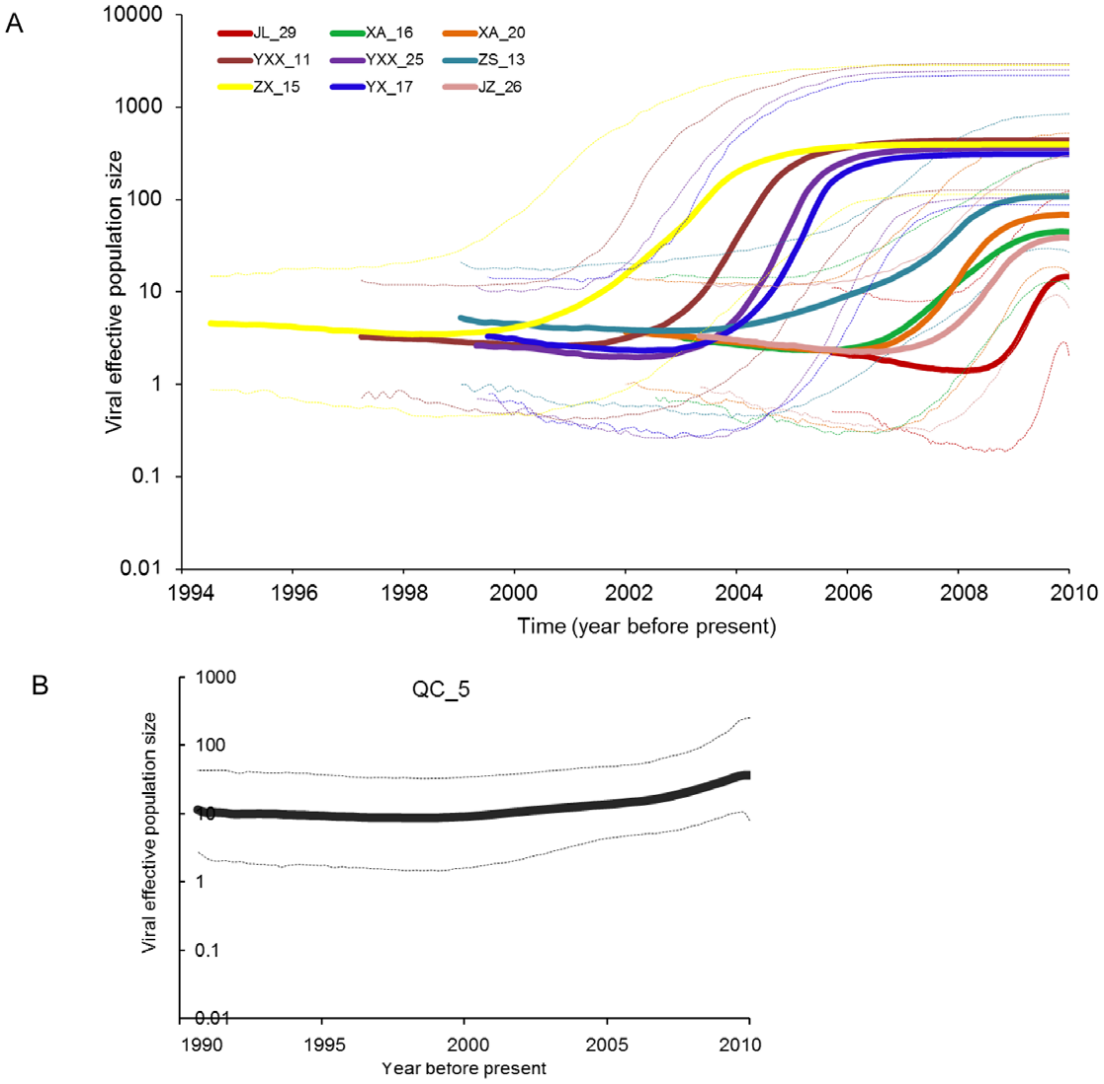
Bayesian Skyline plot depicting GBV-C effective population size in each HIV-infected individual. Recombinant sequences were excluded from the analysis. (A) Viruses in these nine individuals showed three phase growth: stationary phase, followed by sudden increase and stable population size thereafter. (B) Viral population in QC_5 was relatively stable with a sign of recent increase. The substitution rate 3.9×10^−4^sub/site/year that had been previously reported for E gene of GBV-C (Nakao et al., 1997) was used for TMRCA estimation.

**Figure 5:**
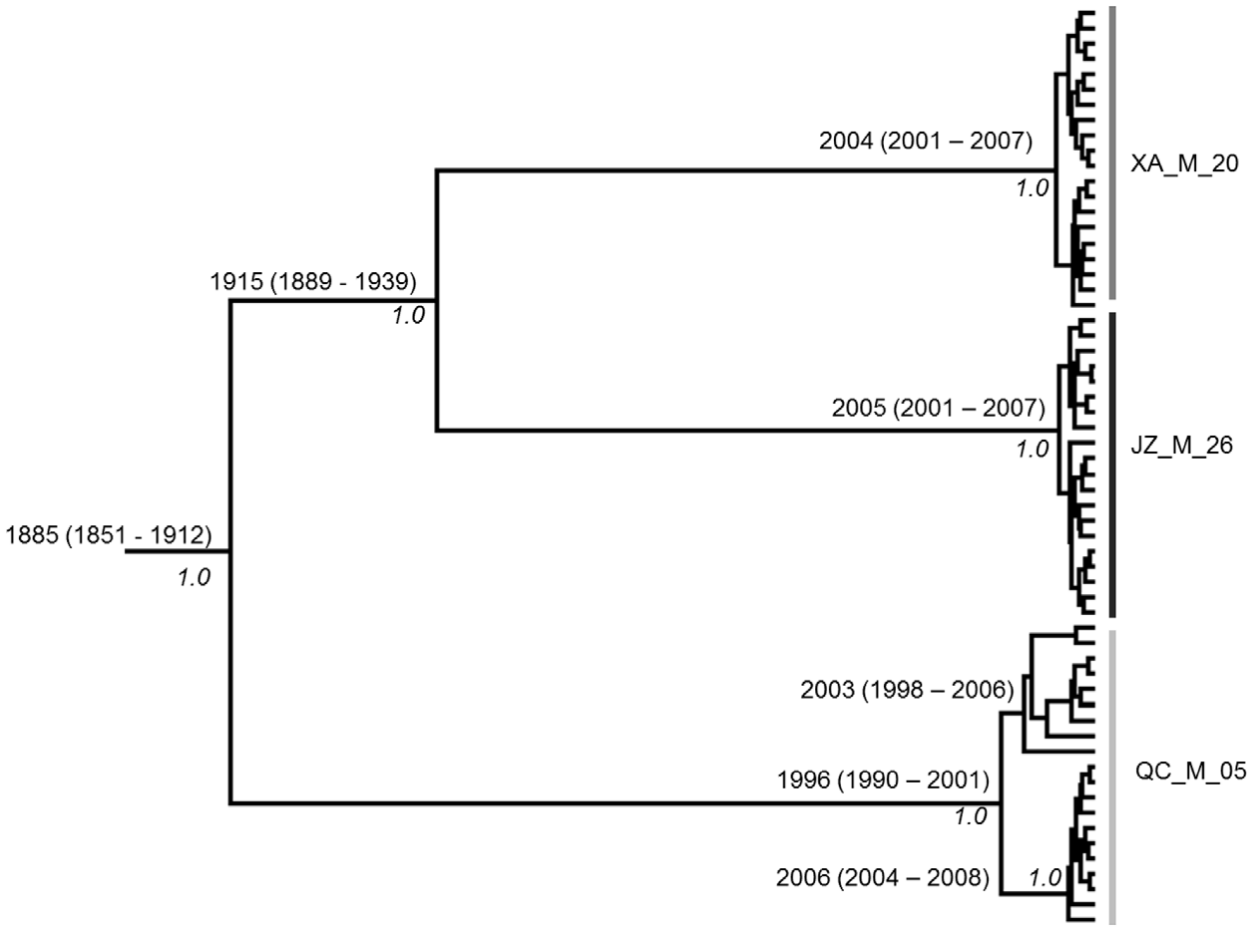
MCC tree showing the estimated time of divergence of GBV-C in QC_M_5, XA_M_20, and JZ_M_26 and the time to the most recent common ancestor. These three groups appeared to be monophyletic in figure 2.

To determine how the pairwise differences among the sequences within each patient were distributed, we performed the mismatch distribution analysis. With the exception of two patients (JZ_26 and QC_5), the observed mismatch histograms for the remaining eight patients were unimodal and the hypothesis of GBV-C viral population expansion within each host couldn't be rejected (p>0.05). While the mismatch histogram in patient JZ_26 declined from a peak of zero difference, the distribution in QC_5 was ragged (Fig. 3C). The L-shape curve in JZ_26 (Fig. 3B) indicated the viral population has recovered from a bottleneck effect followed by sudden population expansion (p>0.05). The ragged distribution of QC_5 suggested that either the viral population within QC_5 was relatively stable or indicated the presence of an admixture of multiple viral populations. To determine how the viral population within each host changed over time, we reconstructed the Bayesian skyline plot (BSP) for each patient (Fig. 4). With the exception of QC_5, the BSP for each patient has revealed three phase growth patterns: a constant population followed by the sudden population expansion and stabilized thereafter. However, the timing of each phase in respective patients seemed to be different (Fig. 4A). Based on the estimation of TMRCAs, viral population in QC_5 was estimated to have diverged approximately during the year 1996 (95% HPD: 1990–2001) and relatively was the oldest (Table 3). Unlike other viral populations, viral population in QC_5 was shown to be relatively stable followed by a steady increase (Fig. 4B). GBV-C sequences from patients XA_M_20, QC_M_05, and JZ_M_26 appeared to form a monophyletic group with strong bootstrap support (Fig. 2), thus allowed us to employ the Bayesian coalescent approach to estimate the time of divergence among the viral lineages in these three patients. GBV-C viral strains in patients XA_M_20 and JZ_M_26 shared a common ancestor and estimated to have diverged approximately during the year 1915 (95% HPD: 1889–1939). The two male patients XA_M_20 and JZ_M_26 infected with HIV through heterosexual and homosexual route respectively, the CD4 cell counts were 203 and 237 cells/µL respectively, and the HIV loads of them were under detection baseline. The TMRCA for all the three viral lineages was estimated as the year 1885 (95%HPD: 1851–1912) (Fig. 5). The dN/dS for each viral population was less than one (Table 3), indicating that purifying selection was the dominant force in the evolution and divergence of GB virus C within respective hosts. To determine whether any of the amino acid sites in E2 gene in each patient are under positive selection, we performed site-specific substitution analysis. The hypothesis of neutral evolution could not be rejected by the LRT (Table 4), thus indicating none of the amino acid sites in each patient are under positive selection.

**Table 4:** Likelihood ratio tests (LRTs) for positive selection.

Patient ID	M7 vs M8(2Δ*l*)	p-value	Positively selected site
JL_M_29	0.00004	1.000	None
QC_M_5	4.99305	0.082	None
XA_M_20	0.00040	1.000	None
YX_M_17	1.66506	0.435	None
YXX_F_25	1.11777	0.572	None
YXX_M_11	0.00002	1.000	None
ZS_M_13	3.02189	0.221	None
JZ_M_26	0.08521	0.958	None
XA_M_16	0.00002	1.000	None
ZX_M_15	0.01574	0.992	None

LRTs revealed none of the amino acid sites of E2 in each patient was under positive selection at p<0.05.

2*Δl*: Differences between the likelihood scores of null (M7) and alternative model (M8).

The p-values were calculated with degrees of freedom = 2.

Null model (M7) that assumes neutral evolution was compared with the model (M8) that assumes positive selection.

## Discussion

The present study investigated the prevalence and population dynamics of GB virus C in HIV infected individuals representing 13 geographic regions in Hubei Province of China. Intravenous drug abuse, paid blood donation, and unsafe sex practice (hetero sexual and homo sexual) are the major route of HIV transmission among the susceptible individuals in Hubei Province of China. Since HIV and GBV-C share similar routes of transmission, the GBV-C prevalence among the HIV infected populations were common and reported to be within a range of 17–41% [18]. According to the present study, 36.5% of HIV-1 infected carriers were concurrently infected with GBV-C. With the exception of two patients, the GBV-C viral strains in the rest eight patients belong to genotype 3, indicating the dominance of genotype 3 in the region. Consistently, previous studies also reported the dominance of genotype 3 in China [19], [20], [49].

Utilizing the full length E2 sequence data and employing the coalescent-based phylogenetic approaches, we have investigated the dynamics of GBV-C in HIV infected subjects. The analysis has revealed the existence of recombinant sequences in two patients. Previous studies have demonstrated that recombination occurs within and between GBV-C genotypes [9]. Thus suggesting recombination force played an important role in the evolution and divergence of GBV-C. Given the convincing role of recombination force in GBV-C viral diversity, the utility of GBV-C viral sequences as the genetic marker to track ancient human migration may yield misleading conclusion if the recombinant sequences were not handled with caution. Patient-wise, clustering of GBV-C within a small geographic region suggested that either the virus has been replicating in the respective hosts for a long period of time or has been evolving at a very high mutation rate within each host. The level of heterogeneity of the virus population within a particular patient was, however, dependent not only upon on the mutation rate of the virus, but also on the viral fitness (ability to produce infectious progeny), and the extrinsic and intrinsic environment (many aspects of the natural history of infection). Alternatively, it might be attributed to the low level of host immunity against this virus [50], [51].

It is worth to note that patients YXX_M_11 and JL_M_29 clustered together and GBV-C sequences from patient YXX_M_11 were basal to the GBV-C sequences from patient JL_M_29. The observation of low branching pattern, low nucleotide diversity (π) and mean pairwise differences (d) in JL_M_29 indicated that patient JL_M_29 was relatively recently infected and viral population within JL_M_29 was emerged from a founding population (Fig. 2; Table 3). Based on the Bayesian coalescent analyses, the sequences from JL_M_29 were diverged since the year 2008 (95% HPD: 2005–2009) (Table 3) indicating recent emergence of GBV-C viral strains in patient JL_M_29. Our clinical data indicated that the two untreated male patients lived in different region of Hubei Province of China (Fig. 1), patient YXX_M_11 was a paid blood donor and patient JL_M_29 was infected with HIV through heterosexual promiscuity. If GBV-C in patient YXX_M_11 was the founding population of patient 29, there should be multiple individuals within the region who were HIV infected by blood transfusion from patient YXX_M_11.

With exception of two patients (JZ_26 and QC_5), the observed mismatch histograms for the remaining eight patients were unimodal. If a patient had been infected multiple times with distinct viral lineages/genotypes, a bimodal mismatch distribution would have been expected. The unimodal mismatch distribution of these eight patients suggested that it was highly unlikely that they were infected multiple times. The viral population expansion/successful adaptation within the host may depend on the viral resistance to the host immunity. However, in immune compromised individuals, viral population may successfully adapt and expand rapidly without any functional modification of its epitopes. Under such circumstances, the glycoprotein gene unlikely to experience any positive selection, since the virus could easily invade the host cell without any functional modification (without any modification in existing fitness) by amino acid modification in its membrane protein. Alternatively, as a nonpathogenic virus, GBV-C virus could elicit weak host immunity which did not crash the viral population [52], [53]. Thus, the finding of GBV-C E2 gene in each HIV-1 infected patient under intense purifying selection is not surprising. Consistently, previous studies have also reported that intra-host HIV-1 evolution was dominated by purifying selection [54]. Nevertheless, further comparison among the GBV-C sequences from HIV-positive and HIV-negative patients would provide clear insight into the dynamics GBV-C and specifically whether GBV-C in two different infection environments has distinct selection profile.

Patient JZ_M_26 had several identical sequences, which means the viral strains did not acquired more mutation and probably they have recently emerged. On the other hand, this patient also had sequences where the pairwise nucleotide difference between them was more than 26. This means that either the virus was in the patient for a long period of time and the population had crashed and recently emerged from a single source or that the patient was infected multiple times. Unlike other viral populations, viral population in QC_5 was shown to be relatively stable followed by a steady increase (Fig. 5). Based on the estimation of TMRCAs, viral population in QC_5, diverging approximately in the year 1996 (95% HPD: 1990–2001), relatively was the oldest (Table 2). According to the clinical data, this patient was untreated and the number of CD4 cells was about 633 cells/µl, suggesting that the progression of HIV disease was slow. Previous studies reported that persistent GBV-C viremia for five or more years after HIV seroconversion was associated with a significant survival benefit [55], [56]. It was not intuitively clear as to whether patient QC_5 was benefitted for being infected with GBV-C for 10 years. Nevertheless, further experiment was required to test whether the stable GBV-C viral population has beneficial effect on the HIV disease progression. In addition, patient QC_5 was detected anti-E2 antibody in the serum, previous studies suggested that the presence of antibody to GBV-C glycoprotein E2 is also associated with survival among those without HIV-1 viremia [11], thus, the presence of GBV-C E2 antibody may has beneficial effect on the progress of HIV disease.

In conclusion, the finding of patient-specific unique GBV-C viral lineage and the evidence of rapid population expansion of the viral lineages in respective HIV-1 infected patients suggested that HIV-1 was unlikely to have any inhibiting effect on the GBV-C viral replication. The finding of within host GBV-C recombinant sequences indicated recombination was one of the significant forces in the evolution and divergence of GBV-C. The lack of the signature of positive selection on the GBV-C E2 sequence was not surprising because GBV-C might have successfully invaded the immune-compromised host without any functional modification by the alternation of amino acid at its membrane protein in order to adapt the new environment.
